# Local injection of adipose-derived mesenchymal stem cells in silk fibroin solution on the regeneration of lower esophageal sphincter in an animal model of GERD

**DOI:** 10.3389/fcell.2023.993741

**Published:** 2023-04-03

**Authors:** Daxu Zhang, Zhanbo Wang, Lianjun Ma, Lijuan Xu, Suna Fan, Yinan Su, Xiaonan Shi, Jingjing Hu, Shuo Zhao, WeiLong Li, Enqiang Linghu, Li Yan

**Affiliations:** ^1^ Department of Hepatobiliary Surgery, Chinese PLA General Hospital, Beijing, China; ^2^ Department of Pathology, Chinese PLA General Hospital, Beijing, China; ^3^ Endoscopy Center, China-Japan Union Hospital of Jilin University, Changchun, Jilin, China; ^4^ Department of Gastroenterology, Beijing Tiantan Hospital, Capital Medical University, Beijing, China; ^5^ State Key Laboratory for Modification of Chemical Fibers and Polymer Materials, College of Materials Science and Engineering, Donghua University, Shanghai, China; ^6^ Department of Hepatobiliary Surgery, Chinese PLA General Hospital, Beijing, China; ^7^ The Second Medical Center and National Clinical Research Center of Geriatric Diseases, Chinese PLA General Hospital, Beijing, China; ^8^ Department of Gastroenterology, The First Medical Center of Chinese PLA General Hospital, Beijing, China

**Keywords:** ADSCs, smooth muscular cells, RSF solution, transplant, gerd

## Abstract

Presently, various tissue engineering methods using adult stem cells and biomaterials are being confirmed to regenerate vessels, cardiac muscle, bladder, and intestines. However, there are few studies about the repair of the lower esophageal sphincter (LES) may help alleviate the symptoms of gastroesophageal reflux disease (GERD). This study aims to determine whether Adipose-Derived Stem Cells (ADSCs) combined with regenerated silk fibroin (RSF) solution could regenerate the LES. *In vitro*, the ADSCs were isolated, identified, and then cultured with an established smooth muscular induction system. *In vivo*, in the experimental groups, CM-Dil labeled ADSCs or induced ADSCs mixed with RSF solution were injected into the LES of rats after the development of the animal model of GERD respectively. The results showed that ADSCs could be induced into smooth muscular-like cells with the expression of h-caldesmon, calponin, α-smooth muscle actin, and a smooth muscle-myosin heavy chain *in vitro*. *In vivo*, the thickness of LES in the experiment rats was much thicker than those in the controlled groups. This result indicated that ADSCs mixed with RSF solution might contribute to the regeneration of the LES, thus reducing the occurrence of GERD.

## 1 Introduction

Over the past 40 years, Gastroesophageal reflux disease (GERD) has spread from western countries to various parts of the world, seriously affecting the quality of life of patients. Obesity, low esophageal sphincter injury, neuromuscular dysfunction, and esophageal fibrosis are the pathological mechanism of this disease (Boeckxstaens et al., 2014). Since the introduction of PPI-dominated treatment, the clinical management of GERD has markedly changed. However, reflux symptoms in some GERD patients may be caused by weak or non-acid reflux, not only acid-related, which has led to research into other drugs, or minimally invasive surgery. Recent studies showed that endoscopy-associated procedures could not reverse the damaged LES function (Akiyama et al., 2018). Therefore, there is an urgent need to find one alternative therapy for GERD (Pasricha et al., 2009; Badylak et al., 2011). The latest studies showed that multipotent stem cells could give rise to smooth muscle cells and contribute to the regeneration of smooth muscles including vascular vessels, bladder, pylorus, and small intestine, which suggested that stem cells might contribute to the regeneration of Lower Esophageal Sphincter (LES) and restore its function (Liu et al., 2015; Gong et al., 2016; Wang et al., 2017; Rahbarghazi et al., 2019). In addition to seed cells, the artificial niche is also a very important factor for the regeneration of smooth muscles.

Several reports showed that induced smooth muscle cells can be from Embryonic Stem Cells (ES), Induced Pluripotent Stem Cells, and Adult Stem Cells (ASCs) (Magli et al., 2016; Saito et al., 2017; Bajek et al., 2018). Although ES and iPS cells retain a higher differentiation potential, their use was limited by ethical issues, the formation of teratomas, etc., (Lee et al., 2017). Adipose-Derived Stem Cells and Bone Marrow derived Stem Cells (BMSCs) (ADSCs) are the major ASCs, which could overcome the above shortcomings and show a high potential capability to smooth muscle cells. Our previous studies showed that ADSCs have similar differentiation capabilities to BMSCs *in vitro* (Xu et al., 2017a). Furthermore, compared with BMSCs, ADSCs have several advantages, which include ease of isolation and expansion, anti-inflammatory properties, and immuneoprivileged status. Based on the previous studies, ADSCs were used as seed cells for the regeneration of LES in this study.

Scaffolds also play an important part in simulating the extracellular microenvironment that might contribute to the regeneration, repair, or replacement of malfunctioning tissues. Silk fibroin is a natural biomaterial obtained from *Bombyx mori* (B. *mori*) cocoons, including more than 90% of the amino acids glycine, alanine and serine (Vepari and Kaplan, 2007). Current studies indicated that regenerated silk fibroin (RSF) contributed to urethra regeneration (Xie et al., 2014), bladder regeneration (Chung et al., 2014), vessel reconstruction (Soffer et al., 2008), cartilage reconstruction (Chao et al., 2010), and peripheral nerve repair (Ki et al., 2015). Our research shows that RSF has good biocompatibility and can be completely degraded within 2–6 months *in vivo* (Xie et al., 2014), and ADSCs-laden RSF could contribute to the liver function of carbon tetrachloride-induced fulminant hepatic failure mice models (Xu et al., 2017b). The above studies suggested that RSF was an ideal biomaterial for organ regeneration.

Several studies reported that RSF seeded with ASCs could promote the repair of skin bone (Ruan et al., 2018), cartilage (Luo et al., 2015), inner ear (Yin et al., 2022), spinal cord (Deng et al., 2021), and bladder (Xiao et al., 2021) regeneration. However, no reports showed the therapeutic potential of RSF seeded with ADSCs on an esophageal injury. In this study, we established a reflux animal model induced by esophagojejunostomy and focused on the ADSCs-based tissue-engineering material for LES regeneration. Our study proved that ADSCs could be induced into smooth muscle-like cells *in vitro*, and the ADSCs or ADSCs-induced laden RSF solution could contribute to the regeneration of LES.

## 2 Materials and ideas

### 2.1 Experimental male rats and the establishment of the model of GERD

Three-day-old and six-week-old male rats (Charles River, China) were purchased from the Laboratory Animal Center of the Academy of Military Medical Sciences of China (Beijing, China). This study has been approved by the Ethics Committee of Animal Facilities of the General Hospital of the Chinese People’s Liberation Army and is in accordance with the relevant provisions of the Guidelines for The Care of Experimental Animals. The 3-day-old rats were used to prepare the ADSCs model, and the 6-week-old rats were used to prepare the GERD model.

The operation was performed by Kumagai et al. (Aikou et al., 2013). The rats underwent anesthesia induction after fasting for 24 h before surgery, followed by an intraperitoneal injection of pentobarbital, and a 2 cm incision was made in the middle of the abdominal section. A 4 mm incision was made at the esophagogastric junction, and a section of jejunum was anastomosed at the esophagogastric junction and the distal end of theTreitz ligament 1 cm.

In this experiment, the model rats were divided into experimental groups and a control group, with 5 rats in each group. After the animal models were established, the ADSCs or induced ADSCs mixed in RSF solutions were injected into the LES of the rats respectively, whereas RSF solutions, and simple ADSCs were used as control groups. Start drinking water 12 h after surgery, and start eating food 24 h after surgery. Only laparotomy was performed in the sham operation group.

### 2.2 Preparation of RSF aqueous solution

In general, the silkworm cocoons were degummed and then dissolved in a 9.0 M lithium bromide solution. The solution was dialyzed in deionized water to get rid of the salt after being diluted, centrifugal, and filtered. Lastly, a 13 wt% RSF aqueous solution was established by forced airflow.

### 2.3 ADSCs culture and characterization

The preparation of ADSCs and the characterization were carried out similarly to our previous studies (Chen et al., 2013). Briefly, the adipose tissues from the inguinal fat pad of 3-day-old rats were minced into 1 mm^3^ and then digested in 1 mg/mL collagenase (Sigma) for 1 h at 37°C. Followed by the termination of digestion and filtration, ADSCs were plated at a density of 5 × 10^5^/cm^2^ in a 6 cm dish with an expansion medium. ADSCs were harvested when they reached 90% confluence, and the passage 4 cells were identified by flow cytometry, osteogenic and adipogenic differentiation.

### 2.4 Identification of smooth muscular-like cells *in vitro*

#### 2.4.1 Induction

In ADSCs, 1 ng/mL transforming growth factor-β1 (TGF-β1), 50 ng/mL platelet-derived growth factor-BB (PDGF-BB), and 50 g/mL β-mercaptoethanol were induced in 24-well plates. Then ADSCs were cultured in HCM for 14 days. The negative control group was undifferentiated cells and the positive control group was smooth muscle cells.

#### 2.4.2 Reverse transcription polymerase chain reaction

On day 7, smooth muscle cells were differentiated 10 times. Total RNA was isolated from ADSCs by Trizol reagent (Sigma-Aldrich, St. Louis, MO, United States). Oligo (dT) primers were used for reverse transcription of the first strand cDNA, and 35 cycles of camping (95°C, 10 min; 58°C, 1 min; 72°C, 5 min), PCR with 10 pmmol/L specific primers. The PCR products were detected by 2% agarose gel electrophoresis. The expression levels of calponin, h-caldesmon, α-smooth muscle actin (α-SMA), and myosin heavy chain (MHC) in smooth muscle cells (SMC) were compared using actin as internal standard (30 cycles of amplification). An image analyzer (Uvitec, Warwickshire, UK) was used to quantitatively analyze the products of undifferentiated and differentiated ADSCs. The primer sequences used are listed in Supplementary Table S1.

#### 2.4.3 Wb analysis

Western blot analysis is described above. In summary, total cellular proteins were prepared by the Bradford method and quantified. 10% sodium dodecyl sulfate-polyacrylamide gel electrophoresis was used, and 80 μg of lysate was electrophoresed on a nitrocellulose membrane (Immoblin-P, Millipore, Bedford, MA, United States). Sealing film at room temperature for 2 h with 5% fat-free milk powder and incubation overnight with Smoothelin (Mouse Monoclonal Antibody, sc-376902, Santacruz, America), MYH11 (Mouse Monoclonal Antibody, sc-6956, Santacruz, America), calponin-1(Rabbit Monoclonal Antibody, 18719S, cell signaling technology, America), caldesmon-1(Rabbit Monoclonal Antibody, 12503S, cell signaling technology, America), α-smooth muscle actin (Rabbit polyclonal Antibody, 14968S, cell signaling technology, America), at 4°C overnight. Wash 3 times 15 min in Tris buffer saline containing Tween 20 (TBST), the goat anti-rabbit IgG antibody coupled with horseradish peroxidase (HRP), rabbit anti-rabbit IgG antibody coupled with HRP (ZB-2306, Zhongshan Jinqiao, Beijing, China) or goat anti-mouse IgG antibody (ZF-0312, Zhongshan Jinqiao, Beijing, China) were incubated at room temperature for 2 h. The membrane was cleaned again in TBST. Add enhanced chemiluminescence reagent to monitor color or anti-β-actin antibody.

#### 2.4.4 Immunofluorescence technique

The cultured cells were fastened with 4% paraformaldehyde (Sigma-Oldridge) at room temperature for 30 min, washed twice with phosphate buffer solution (PBS), and infiltrated with 1% Triton X-100 (Sigma-Oldridge) at room temperature for 20 min. The cells were then incubated at room temperature for 2 h with a blocking solution composed of PBS and 10% normal goat serum NGS. Immunofluorescence staining, primary antibody calcium bridge. For immunofluorescence staining, primary antibodies caldesmon (1:200, Rabbit Monoclonal Antibody, ab32330, abcam, America), calponin (1:100, Mouse Monoclonal Antibody, sc-58707, Santacruz, America), MHC (1:100, Goat polyclonal Antibody, sc-1592 9, Santacruz, America Antibody, sc-58707, Santacruz, America), alpha smooth muscle actin (1:200, Rabbit Monoclonal Antibody, ab124964, Abcam, America), Smoothelin (1:100, Mouse Monoclonal Antibody, sc-376902, Santacruz, Ameri ca) were used. The primary antibody was incubated overnight at 4 °C, and the secondary antibody was incubated at 37°C for 2 h. The 4′,6-diamidino-2-phenylindole were stained at room temperature for 5 min and photographed with a structurally illuminated fluorescence microscope.

### 2.5 CM-Dil staining and cell tracing

Passage 3 and passage 4 ADSCs or induced ADSCs were labeled with CM-Dil (Invitrogen Operate according to the instruction) and then mixed with RSF solutions respectively. At different times of 2 weeks and 4 weeks after transplantation, the located cells in the LES of the rats were traced by small animal imaging techniques (Berthold Technologies LB 983 NC 100).

### 2.6 Necropsy and tissue processing

Rats were anesthetized with pentobarbital sodium at the 2nd, 4^th,^ and 6th weeks, perfused with PBS (pH 7.4) through the left ventricle for 1 min, and buffered with 4% paraformaldehyde for 10 min. The esophagus and stomach were excised and opened along larger curvature. The esophagus and stomach were cut into strips 2 mm wide parallel to the small curvature. Stored in 4% paraformaldehyde at 4°C for 2 h, some tissues were embedded in tissue-Te OCT complex (Tokyo Cherry, Japan), frozen in liquid nitrogen, and embedded in paraffin to prepare 4-um sections.

### 2.7 Follow-up of the survival rate of the experimental rats

ADSCs or induced ADSCs mixed with RSF solution were injected into the LES of rats after the establishment of an animal model of GERD respectively as the experimental groups, while simple ADSCs or RSF solution injection were treated as controlled groups (*n* = 8). Within 48 h, one rat died in the RSF group, two rats died in the ADSCs group, two rats died in the ADSCs-RSF group, and one rat died in the induced ADSCs-RSF group. No animals died later than.

### 2.8 Statistical analysis

The data are expressed as mean ± SD. The student’s test of esophageal thickness was performed using SPSS21.0 (SPSS Inc., Chicago, IL, United States) software. All statistical analyses were *p* < 0.05. The comparison data between groups had obvious statistical significance.

## 3 Conclusion

### 3.1 Cytomorphology and phenotypic characterization of ADSCs

**FIGURE 1:**
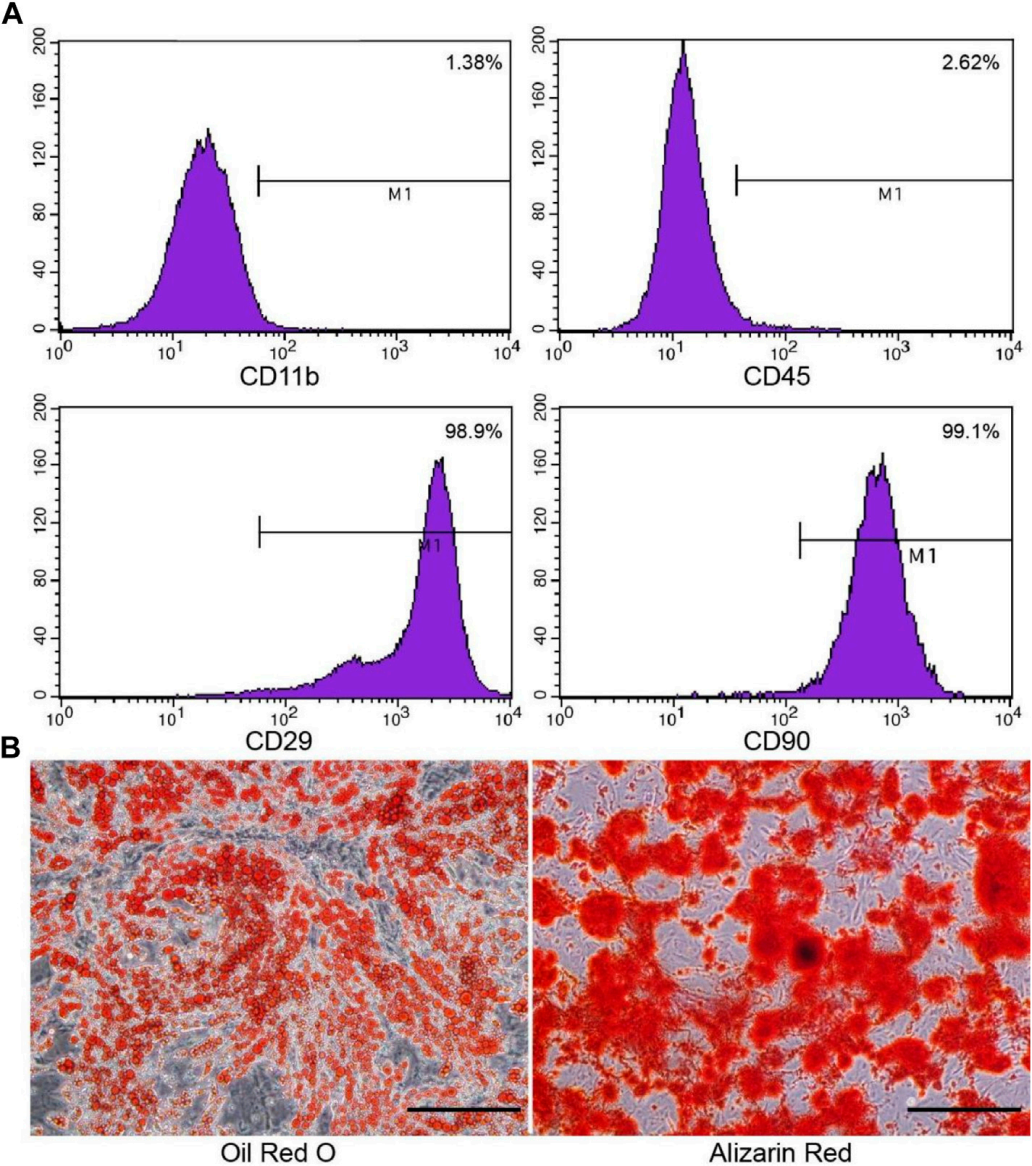
Phenotypic characterization of the cultured ADSCs by flow cytometry and staining. **(A)** ADSCs express CD90 and CD29, but do not express CD11b or CD45, which is consistent with stem cells. **(B)** Oil-Red-O staining was used to identify adipogenic differentiation, and alizarin red was used to identify osteogenic differentiation. Scale bar = 200 μm.

Cell morphology was observed under an inverted microscope. ADSCs were rarely observed on day 2 of primary culture. The number of cells increased on day 3 (cell density was about 70%). On the 4th day, the cells showed a spiral structure, reaching 90% confluence. Cells were passaged at 1:3, and ADSCs were fusiform from passages 1 to 4. The surface markers of ADSCs were analyzed by flow cytometry. ADSCs convey stem cell-related surface markers CD90 and CD29 but do not convey CD11b or CD45 (Figure 1A).

The second and third generations evaluated the adipogenic and osteogenic differentiation of ADSCs. On day 3, small round vacuoles began to appear in the cytoplasm. On day 8, most of the induced cells appeared with many lipid droplets, and the cells became round, oval, or polygonal. Oil-red-O staining identified fat differentiation, lipid vacuoles were bright red. After 3 weeks of induction, cells gathered in some areas, forming a multi-layer, nodular structure, known as bone nodules. Identification of osteogenic differentiation by alizarin red staining (Figure 1B).

### 3.2 Identification of smooth muscle-like cells *in vitro*

**FIGURE 2:**
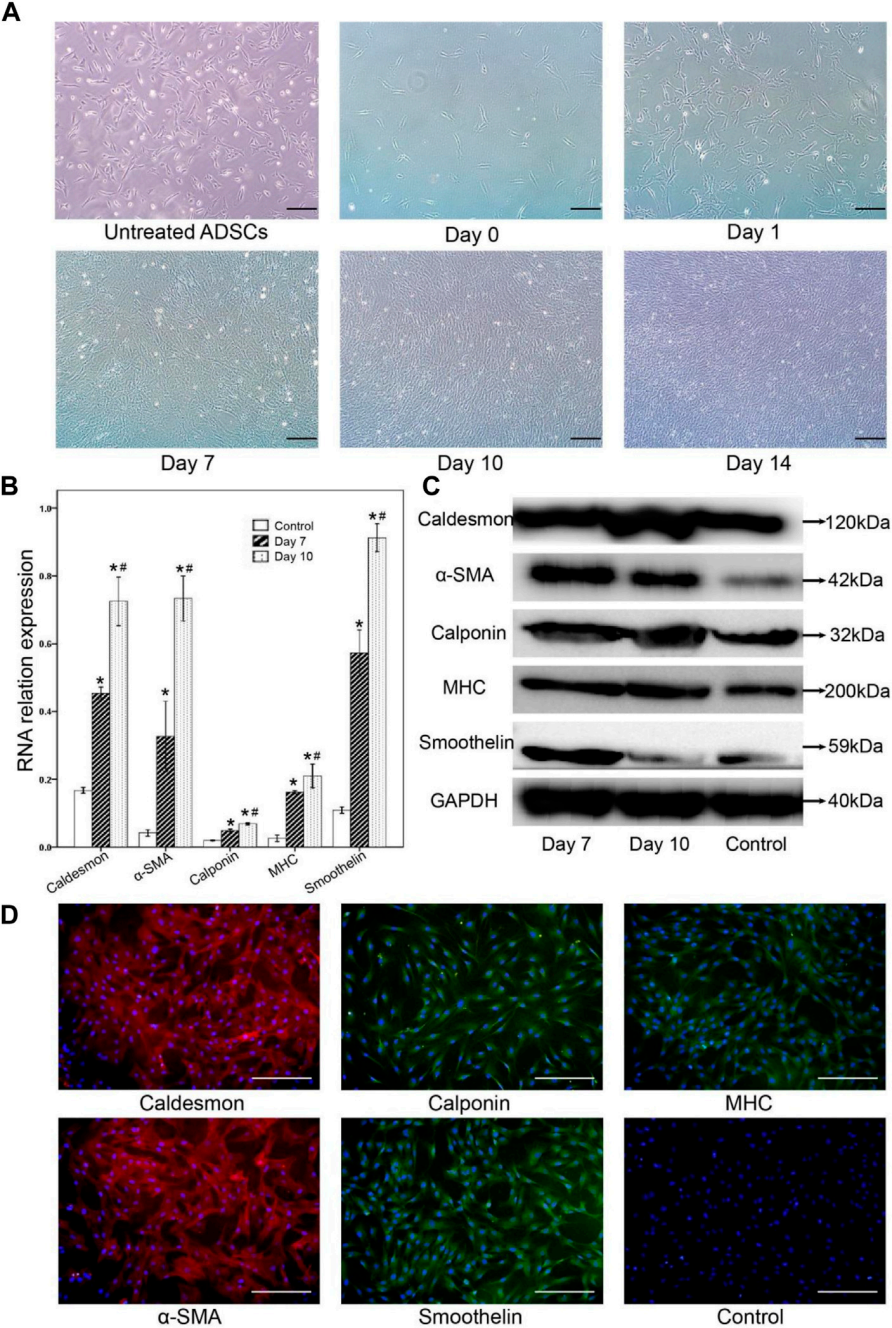
The morphological changes of adipocytes and the changes of smooth muscle-related markers after muscle differentiation were observed by RT-PCR, Western blot analysis, and immunofluorescence staining. **(A)** After muscle tissue differentiation, the cell morphology gradually changed. From day 10, the cells were induced to be spindle-shaped, strip-shaped, and fibroblast-like cells, while the untreated ADSCs formed monolayer fibroblast-like cells (control group). **(B)** Gene expression of caldesmon, α-SMA, calponin, MHC, and smoothelin was significantly detected in smooth muscle-like cells on day 7, then enhanced 10 days after muscle differentiation. Undifferentiated cells were set as a negative control.* Compared with the uninduced group, the difference was statistically significant; # Day 10 compared with the day 7 group, the difference was significant. **(C)** Western blot analysis of smooth muscular-related markers in the smooth muscle-like cells on day 7, and day 10, undifferentiated adipose-derived stromal cells (ADSCs) served as a negative control. **(D)** On day 10 after differentiation, immunofluorescence staining showed that differentiated smooth muscle-like cells expressed heavy calmodulin-binding protein, α-SMA, calmodulin, MHC, and smoothelin, while undifferentiated ADSCs did not express smooth muscle-related markers. Scale bar = 200 μm.

Induction of TGF-β 1, PDGF-BB, and β-mercaptoethanol for 2 weeks, the differentiated cells acquired typical smooth muscle cells morphology with spindle-like, elongated, fibroblast-shaped cells (Figure 2A), whereas the non-differentiated control groups formed a monolayer of fibroblast-like cells. On days 7 and 10 after differentiation, the expressions of several smooth muscular genes or proteins were examined by RT-PCR, and Western blot. Uninduced cells served as negative controls. The results of RT-PCR showed that smooth muscle-related genes including h-caldesmon, α-SMA, and smoothelin were significantly enhanced in smooth muscle-like cells, which agreed with the conclusion of Western blot (Figure 2B).

The expression of caldesmon, α-SMA, calponin, MHC, and smoothelin in smooth muscle-like cells was confirmed by Western blot analysis (Figure 2C). The expression of caldesmon, α-SMA, calponin, MHC, and smoothelin increased on the 7th and 10th days in the smooth muscle-like cells compared with undifferentiated ADSCs.

10 days after differentiation, immunoluorescence staining showed that differentiated smooth muscle-like cells expressed calcineurin binding protein, α-SMA and smooth muscle fat, while undifferentiated ADSCs did not express smooth muscle-related markers (Figure 2D).

### 3.3 Identification of animal model of GERD and the location of ADSCs in LES

**FIGURE 3:**
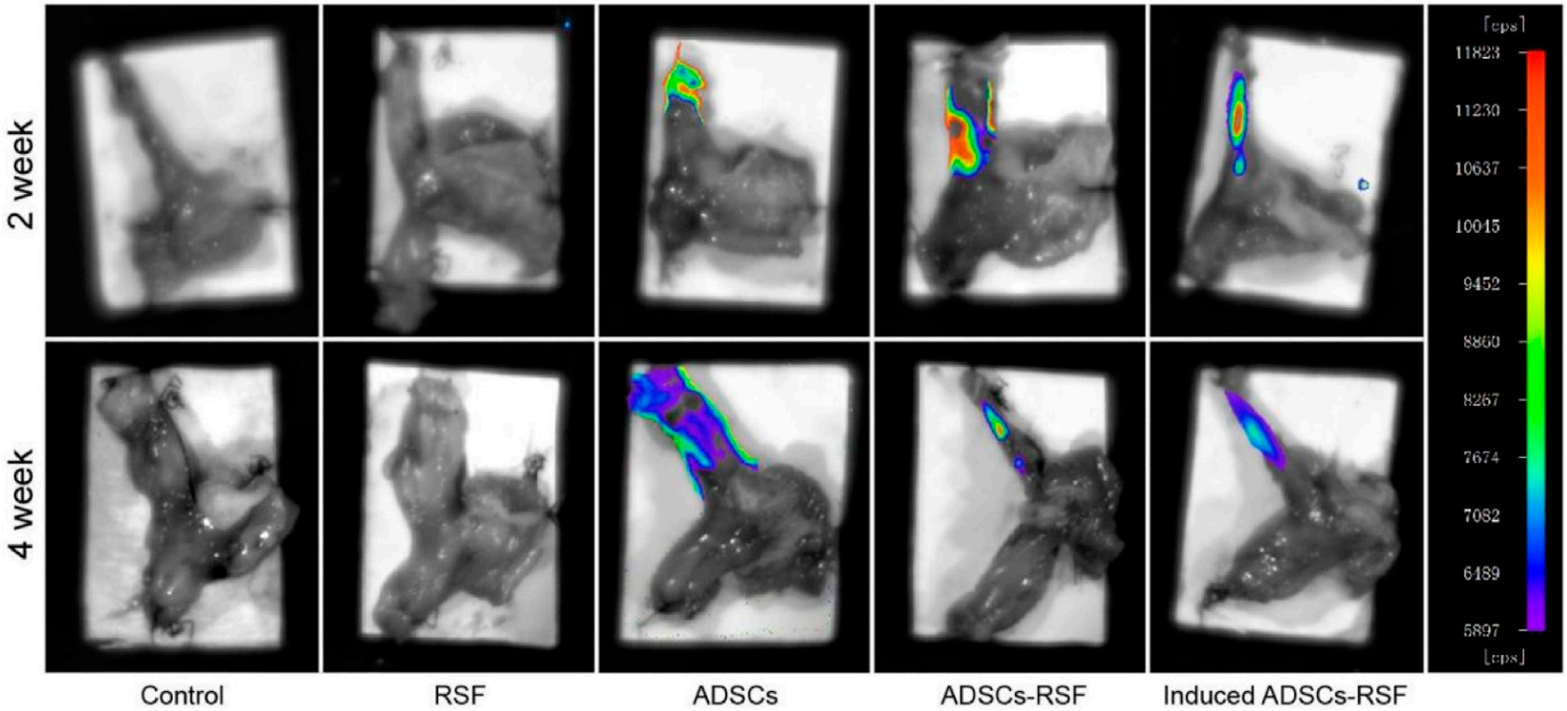
To investigate the engraftment of ADSCs, simple RSF, the ADSCs, the RSF mixed with ADSCs, or RSF mixed induced ADSCs were injected into the LES of rats. Small animal imaging technology observed that ADSCs emitted fluorescence in the second week, and the fluorescence area gradually decreased in the fourth week.

Most rats survived after esophagojejunostomy until sacrifice. HE staining showed that there were no signs of anastomotic stricture, but inflammatory evidence could be observed, which demonstrates that reflux induced by esophagojejunostomy was successfully established. To investigate the engraftment of ADSCs, the ADSCs or induced ADSCs mixed with RSF solution or simple RSF solution were injected into the LES of rats. On weeks 2 and 4, small animal imaging technology was used to observe the fluorescence emitted by ADSCs. With the passage of time, the fluorescence region gradually decreased and disappeared in the sixth week (Figure 3). On the contrary, there was no fluorescence expression in the group of RSF solution.

### 3.4 The thickness of LES between preoperation and postoperation

**FIGURE 4:**
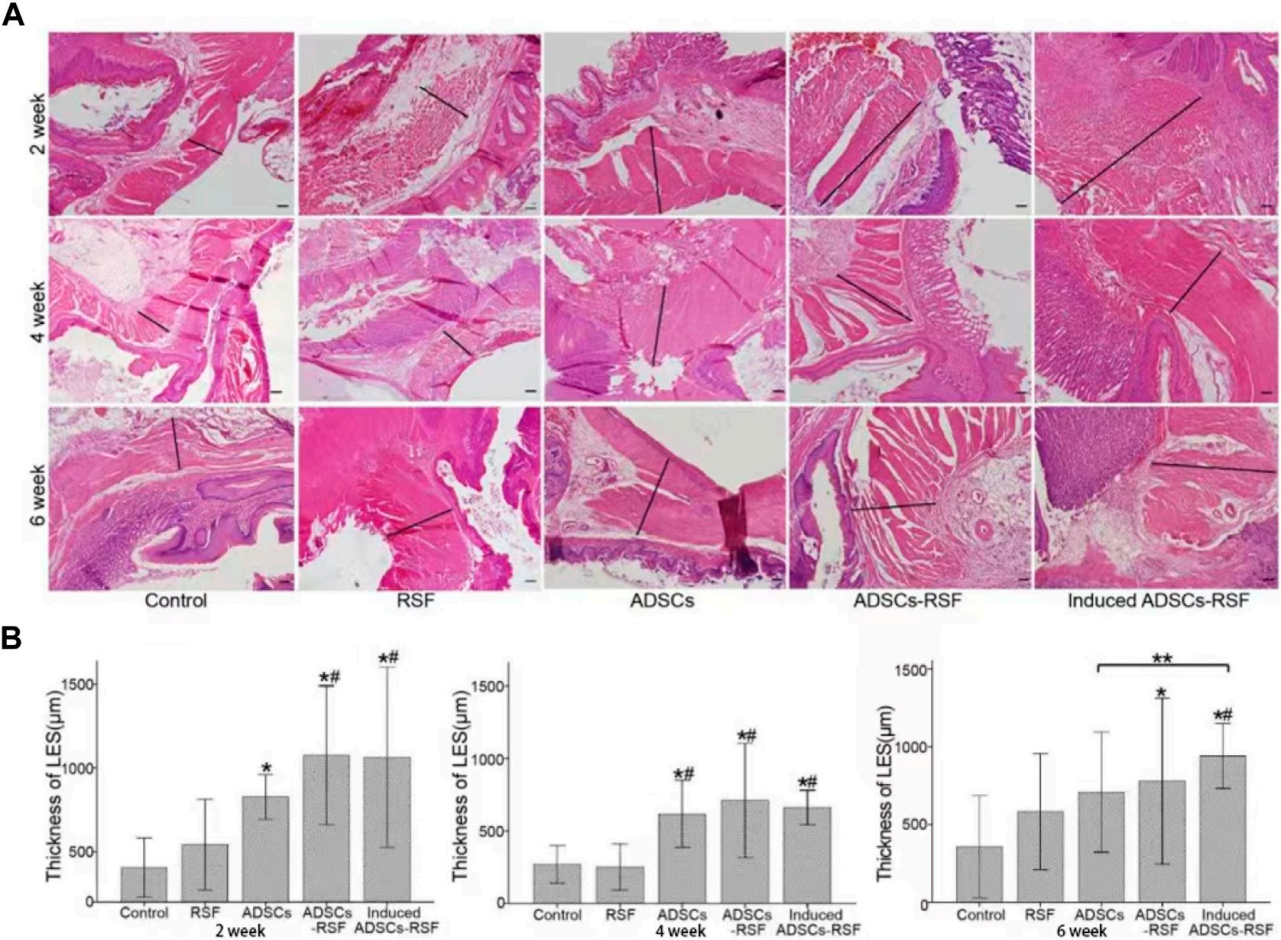
Establish gastroesophageal reflux rat models and transplant PBS, RSF, ADSCs, ADSCs-RSF, and induced ADSCs-RSF into the lower esophagus of the models separately. **(A)** HE staining showed the thickness of the lower esophagus sphincters (LES) measured at 2, 4, and 6 weeks after transplantation. **(B)** According to Statis data, compared with the control group and RSF group, the thickness of LES in the experimental groups increased significantly and more significantly in ADSCs-RSF and induced ADSCs-RSF group. Along with the time going on, compared with the ADSCs group, the thickness of LES in the induced ADSCs-RSF showed statistical differences at 6 weeks after transplantation. * Compared with the control group, the difference was statistically significant; # Compared with the RSF group, the difference was significant; ** The difference between the two groups was statistically sense. Scale bar = 200 μm.

To address whether ADSCs mixed with RSF aqueous could regenerate the LES of the rat model of GERD, RSF aqueous, ADSCs, and ADSCs mixed with RSF aqueous were transplanted to oral side of esophagojejunum anastomosis respectively. After transplantation, the thickness of LES in different group rats was calculated at different time points of week 2, 4, and 6. As result, compared with the saline and RSF group, the thickness of lower esophageal muscles in the experimental groups increased significantly and more significantly in ADSCs-RSF and induced ADSCs-RSF groups. Along with the time going on, compared with the ADSCs group, the thickness of LES in the induced ADSCs-RSF showed statistical differences at week 6 after transplantation. In addition, the inflammatory response showed no evident difference (Figure 4).

## 4 Discussion

Gastroesophageal reflux disease is mainly caused by lower esophageal sphincter (LES) injury. At present, it is common in clinical practice after endoscopy-associated procedures, and very few are caused by esophageal hiatal hernia caused by diaphragm loss. The disease seriously affects the quality of life of patients. At present, the treatment methods based on long-term use of PPI (Chen and Brady, 2019) have problems such as renal dysfunction (Maret-Ouda et al., 2020), *Clostridium difficile* and pulmonary infection (Cao et al., 2018; Nguyen et al., 2020), and osteoporosis (Mortensen et al., 2020). The pathogenesis of gastroesophageal reflux disease is mainly LES injury caused by many factors (Boeckxstaens et al., 2014), which leads to the retrograde entry of digestive juice into the esophagus and causes esophageal mucosal injury. At present, researchers proposed that reconstruction of LES will become a more effective treatment for gastroesophageal reflux disease based on the pathogenic mechanism. Stem cells are a kind of cells with self-proliferation and differentiation ability (Zakrzewski et al., 2019). After stem cells are implanted into the damaged LES, they can repair the structure of the LES and restore its physiological function. Biomaterials can provide a microenvironment similar to the extracellular matrix for the proliferation and differentiation of stem cells (Chen and Liu, 2016), thereby might build an LES regeneration microenvironment to reconstruct the function of LES.

In our previous studies, we have demonstrated that RSF combined with adult stem cells can promote liver reconstruction (Xu et al., 2017b). Also, RSF combined with adult stem cells has the same effect in organs including the bladder (Chung et al., 2014) and urethra (Xie et al., 2014; Xiao et al., 2021). Based on our previous studies, in this study, we first established an induction system for ADSCs to differentiate into smooth muscle-like cells. Then, we verified the biocompatibility between the ADSCs and RSF by HE staining, SEM analysis, and LIVE/DEAD staining *in vitro* and *in vivo*, we found the RSF scaffold could be gradually degraded after 3 months with the reduction of the inflammatory index (Xu et al., 2017c), but without tumorigenicity and coinfection. Finally, after we mixed ADSCs with RSF aqueous solution, and then injected the mixture into the LES of the GERD model, we explored the potential mechanism of ASDCs combined with RSF aqueous solution to promote LES regeneration from two aspects. On the one hand, ADSCs might contribute to the regeneration of the LES of the animal model of GERD, because of the transdifferentiating function of ADSCs in the scaffolds of RSF. on the other hand, based on the volume effect or reactive proliferation of biomaterials, the thickness of LES can be increased to a certain extent, which may contribute to the anti-reflux barrier of LES without side effects and toxicity. In summary, local injection of adipose-derived mesenchymal stem cells combined with RSF may be another method for the treatment of LES injury caused by gastroesophageal reflux disease. In the future, these regenerated smooth muscle materials might be applied in GERD patients caused by endoscopy-associated procedures.

## 5 Conclusion

Our result indicated that ADSCs mixed with RSF solution might be an alternative treatment for LES injury-caused GERD. Thus, local injection of adipose-derived mesenchymal stem cells combined with RSF might contribute to the regeneration of the LES and reduce the occurrence of GERD.

## Data Availability

The raw data supporting the conclusion of this article will be made available by the authors, without undue reservation.
