# Supplementary figures and images for: Local injection of adipose-derived mesenchymal stem cells in silk fibroin solution on the regeneration of lower esophageal sphincter in an animal model of GERD
Source: Front Cell Dev Biol. 2023 Apr 3;11:993741. doi: 10.3389/fcell.2023.993741 (PMC10106618; doi:10.3389/fcell.2023.993741)

Document 1

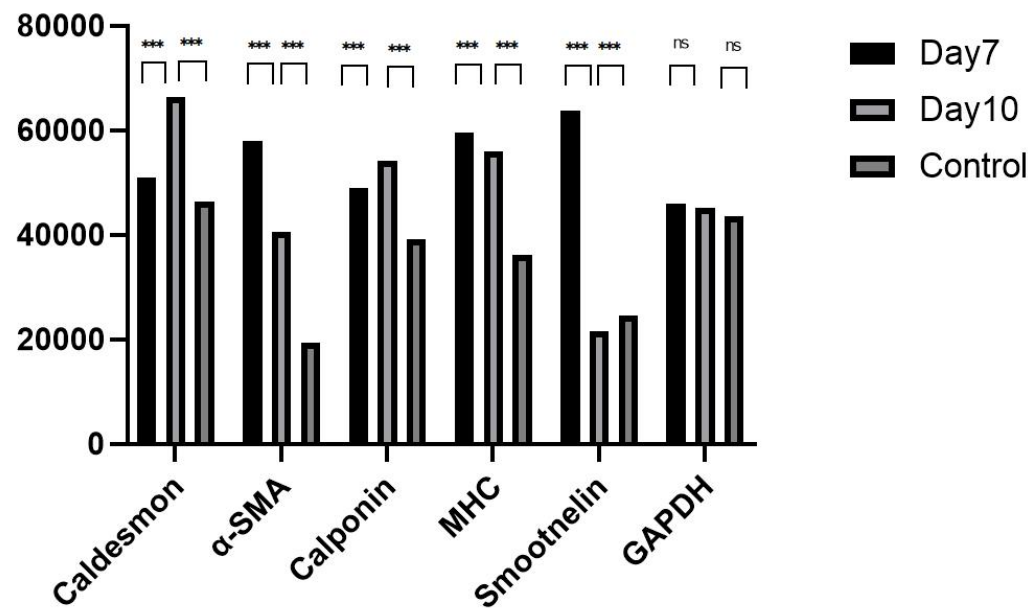

Fig.1. western blot

Document 2

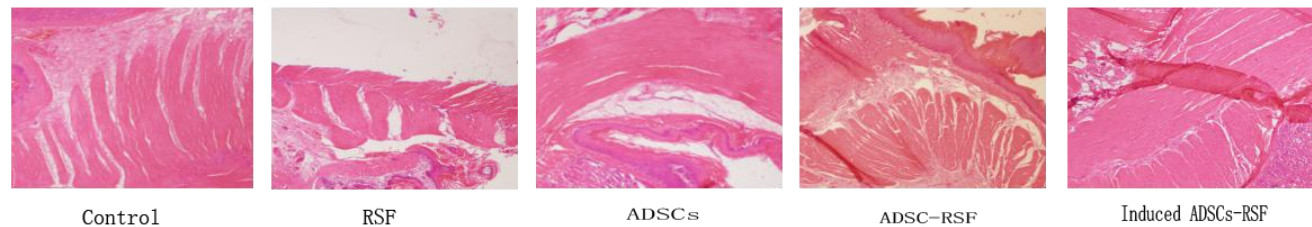

Fig.2. HE staining

Supplement: Supplementary file 1 [file DataSheet1.PDF]
